# A Class II small heat shock protein OsHsp18.0 plays positive roles in both biotic and abiotic defense responses in rice

**DOI:** 10.1038/s41598-017-11882-x

**Published:** 2017-09-12

**Authors:** Jie Kuang, Jianzhong Liu, Jun Mei, Changchun Wang, Haitao Hu, Yanjun Zhang, Meihao Sun, Xi Ning, Langtao Xiao, Ling Yang

**Affiliations:** 1 College of Chemistry and Life Sciences, Zhejiang Normal University, Jinhua, Zhejiang, 321004 China; 2 Hunan Provincial Key Laboratory of Phytohormones and Growth Development, Hunan Agricultural University, Changsha, Hunan 410128 China

## Abstract

Bacterial blight caused by *Xanthomonas oryzae* pv. *oryzae* (*Xoo*) is one of the most devastating diseases of rice. However, the molecular mechanism underpinning the *Xoo* resistance of rice is still not fully understood. Here, we report that a class II small heat shock protein gene, *OsHsp18.0*, whose expression was differentially induced between a resistant and a susceptible variety in response to *Xoo* infection, plays positive roles in both biotic and abiotic resistance. The molecular chaperone activity of OsHsp18.0 was confirmed by a bacterium-expressed glutathione S-transferase fusion protein. Overexpression of *OsHsp18.0* in a susceptible rice variety significantly enhanced its resistance to multiple *Xoo* strains, whereas silencing of *OsHsp18.0* in a resistant variety drastically increased its susceptibility. The enhanced *Xoo* resistance in *OsHsp18.0*-overexpressing lines was positively correlated with the sensitized salicylic acid-dependent defense responses. In addition to disease resistance, the *OsHsp18.0* overexpressing and silencing lines exhibited enhanced and reduced tolerance, respectively, to heat and salt treatments. The subcellular localization study revealed that the green fluorescent protein-OsHsp18.0 was enriched on the nuclear envelope, suggesting a potential role of OsHsp18.0 in the nucleo-cytoplasmic trafficking. Together, our results reveal that the rice OsHsp18.0 is a positive regulator in both biotic and abiotic defense responses.

## Introduction

All organisms produce heat shock proteins (Hsps) in response to elevation in temperature and certain other stresses^1^. The Hsp superfamily is one of the most ubiquitous and evolutionarily conserved proteins across all species, and has been classified on the basis of their molecular weight into five groups^2, 3^. In plants, small Hsps (sHsps) with monomer sizes ranging from 12 to 42 kDa are the most diverse and more abundant than in other organisms, therefore suggesting that they may play important roles in plant stress tolerance and other cellular processes under normal conditions^2–4^. sHsps share a signature C-terminal alpha-crystallin domain (ACD) of ~90 amino acids, which has a conserved β-sandwich structure^4, 5^. Under *in vitro* conditions, sHsps are often found to assemble into large oligomers of 12–40 subunits, utilizing dimers as the building block^6^. sHsps do not require ATP to bind substrate proteins and they have a very high capacity for binding denatured substrates^7, 8^. Some sHsps have been demonstrated to form complexes with denatured proteins and prevent their aggregation *in vitro* and *in vivo* ^2, 9, 10^. From these complexes, the target proteins are subsequently refolded by Hsp100/Hsp70 and cochaperones in an ATP-dependent manner during the recovery phase^6, 8, 11^.

Plant sHsps are all encoded by nuclear genes and can be further divided into at least 16 subfamilies based on amino acid sequence similarity and localization to distinct subcellular compartments^12^. Eleven subfamilies, class I (CI) to CXI are present in the cytoplasm and/or nucleus, while the others are targeted to the plastids, mitochondria, peroxisomes, and endoplasmic reticulum^4, 12^. The CII subfamily has a conserved N-terminal amino acid motif (DA-AMAATP) that is not found in the other cytoplasmic/nuclear sHsps^7^. Compared to CI sHsps, fewer reports were focused on the function of CII sHsps in plants. In rice, only two out of 23 sHsps, OsHsp18.0 and OsHsp17.8, were categorized into the CII subfamily^4^. It should be noted, the *OsHsp18.0* gene has been functionally characterized only to a limited degree^13, 14^. Heterologous expression of the OsHsp18.0 fusion protein increased thermotolerance of *Escherichia coli* cells *in vivo* and provided thermoprotection to *E. coli* soluble proteins *in vitro* ^15^. However, the roles of *OsHsp18.0* in disease resistance as well as in abiotic stress have not been investigated extensively.

Plants respond to pathogen infection through two types of immune responses: basal and isolate-specific disease resistance. The basal defense response is activated by virulent pathogens through the interaction of host pattern-recognition receptors and pathogen-associated molecular patterns (PAMPs). The isolate-specific or gene-for-gene defense response is triggered by host nucleotide-binding (NB) - leucine-rich repeat (LRR) type resistance (R) proteins recognizing isolate-specific pathogen effectors^16^.

Bacterial blight caused by *Xanthomonas oryzae* pv. *oryzae* (*Xoo*) is one of the most devastating bacterial diseases in rice^17^. Accumulating evidence has revealed that the molecular mechanisms of rice qualitative resistance to *Xoo* are largely different from those of R protein-mediated resistance or effector-triggered immunity (ETI) in other plant–pathogen pathosystems^18^. While 21 out of 22 cloned *R* genes against *Magnaporthe oryzae* encode NB-LRR type proteins, only one out of seven cloned *Xoo R* genes encodes this type of protein; although the rice genome contains 623–725 NB-LRR genes^19^. The rest of six cloned *Xoo R* genes encode different types of proteins, indicating the functional diversity in rice–*Xoo* interactions^18^. Given the importance of the rice *Xoo* disease, there is an urgency to clone more *Xoo R* genes to aid in fully understanding the molecular mechanisms underpinning the resistance against *Xoo*.

To identify the genes that are potentially engaged in *Xoo* resistance, microarray analysis was performed on the Affymetrix Rice Genome Genechip Array using RNA probes isolated from rice (SH5) leaves inoculated with *Xoo* strain Zhe173. Among the upregulated genes, the expression of a gene encoding 18-kDa CII heat shock protein (designated *OsHsp18.0*) was induced 3.6-fold during the incompatible interaction. We confirmed that bacterium-expressed OsHsp18.0-glutathione S-transferase (GST) fusion protein possessed activity of a molecular chaperone. In addition, our transgenic studies indicated that OsHsp18.0 played positive roles not only in both PAMP-triggered immunity (PTI) and ETI or qualitative resistance but also in heat and salt tolerance. Subcellular localization analysis revealed that OsHsp18.0 was predominantly localized in the cytoplasm and especially enriched on the nuclear rim, suggesting a potential role of OsHsp18.0 in the nucleo-cytoplasmic trafficking. Together, our results reveal that *OsHsp18.0* plays positive roles in both *Xoo* resistance and abiotic stress tolerance in rice. The possible mechanism by which OsHsp18.0 enhances *Xoo* resistance and heat/salt tolerance is discussed.

## Results

### *OsHsp18.0* is differentially induced between a resistant and a susceptible variety in response to *Xoo* infection

**Figure 1:**
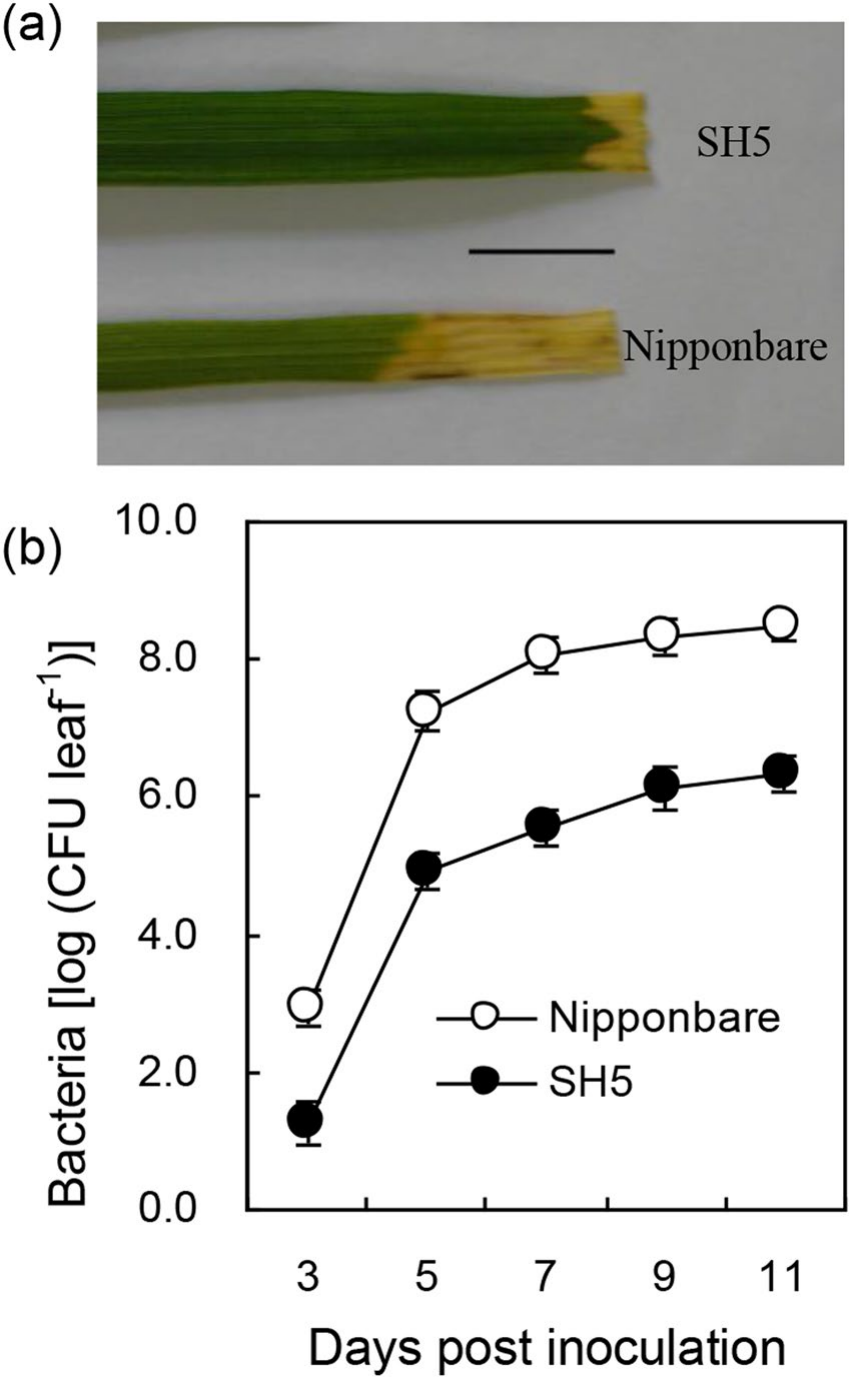
Differential responses of SH5 and Nipponbare to *Xoo* infection. (**a**) Comparison of lesion length formed on the leaves of SH5 and Nipponbare at 14 dpi. Five-leaf stage seedlings were inoculated with *Xoo* strain Zhe173 by clipping method. The experiment was repeated three times with similar results. Scale bar, 1 cm. (**b**) Growth curves of *Xoo* strain Zhe173 on the leaves of SH5 and Nipponbare. Bacterial growths were determined from three infected leaves at each time point by counting CFU. Error bars stand for standard deviation (SD).

Rice variety SH5 is resistant to the *Xoo* strain Zhe173, whereas Nipponbare is susceptible. Our infection results showed that the lesion length formed on the leaves of SH5 in response to Zhe173 infection was significantly shorter than that formed on the leaves of Nipponbare, 0.4 ± 0.1 cm for SH5 *vs* 1.7 ± 0.2 cm for Nippobare at 14 days post inoculation (dpi) (Fig. 1a). Consistently, the bacterial growth rate on the leaves of SH5 was significantly lower (*p* < 0.01) than that on the leaves of Nipponbare at 3 to 11 dpi of *Xoo* (Fig. 1b).

To identify the induced genes in SH5 by *Xoo* strain Zhe173, microarray analysis using the Affymetrix Rice Genome Genechip (Shanghai Outdo Biotech Co., Ltd., China) was performed using RNA purified from leaves of SH5 either at 0 or 12 h post inoculation (hpi). Microarray analysis revealed that one (AK071240) of genes was induced 3.6 fold in response to Zhe173 infection relative to mock treatment. The cDNA sequences amplified from both resistant and susceptible varieties were identical, which was 733 bp in length and contained a 501-bp ORF encoding 166 amino acids. The ORF sequence showed 100% identity with *OsHsp18.0* (DQ180746)^13^ therefore it is still referred to as *OsHsp18.0* (for *Oryza sativa* small heat shock protein 18.0).

**Figure 2:**
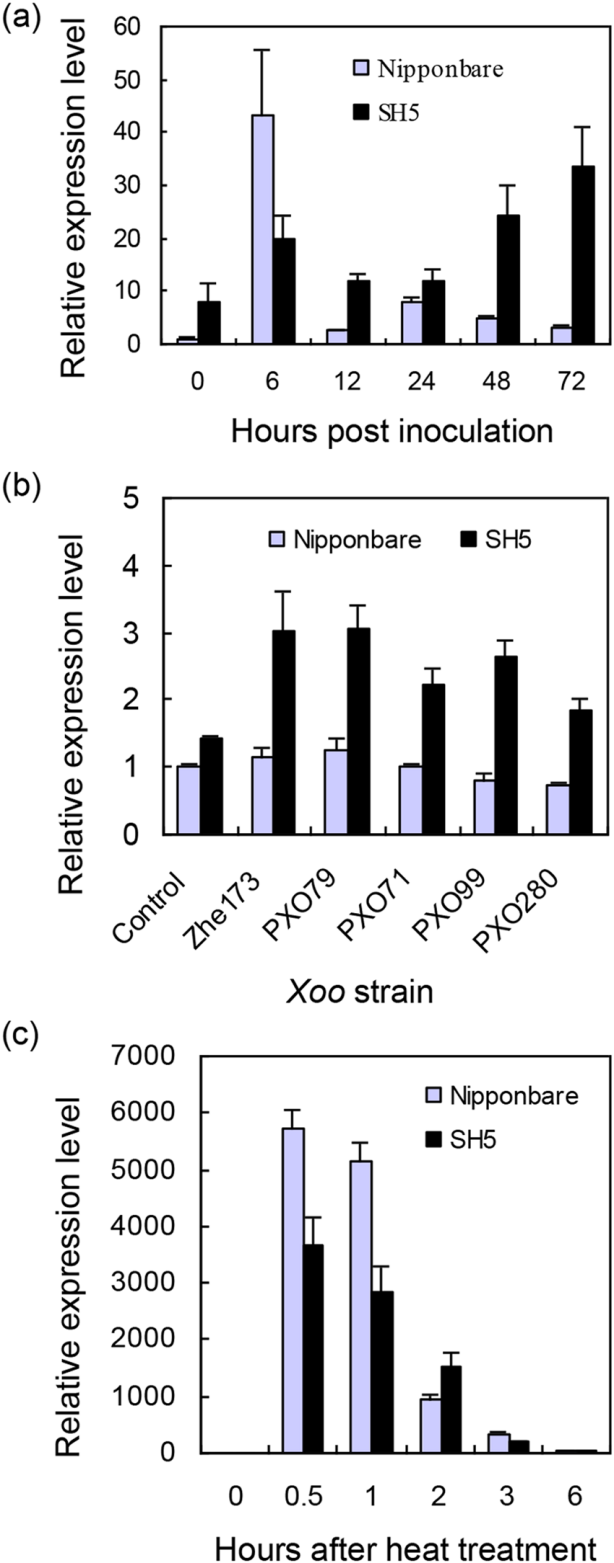
Expression patterns of *OsHsp18.0* in SH5 and Nipponbare in response to *Xoo* infection and heat treatment. (**a**) The relative expression of *OsHsp18.0* in SH5 and Nipponbare in response to *Xoo* strain Zhe173 infection. qRT-PCR was performed using total RNA isolated from inoculated plants at five-leaf stage. The expression level (arbitrary units) was normalized using *β*-actin as an internal reference, and the normalized expression level of *OsHsp18.0* in Nipponbare under non-infected condition at 0 h was set as 1. (**b**) The relative expression of *OsHsp18.0* in SH5 and Nipponbare in response to five *Xoo* strains at 5 dpi. Normalized mRNA level in Nipponbare at 0 d was arbitrarily set as 1. (**c**) The relative expression of *OsHsp18.0* in rice leaves of Nipponbare and SH5 after treated with 45 °C. The normalized expression level in leaves of Nipponbare at 0 h was set as 1. Bars represent means (three replicates) ±SD.

*OsHsp18.0* expression patterns between SH5 and Nipponbare in response to the *Xoo* infection were further compared by qRT-PCR. In the absence of *Xoo* infection, the expression level of *OsHsp18.0* in SH5 was higher than in Nipponbare (Fig. 2a). In response to the infection of *Xoo* strain Zhe173, the induction of *OsHsp18.0* was initially higher in Nipponbare than in SH5 at 6 hpi. However, the induction of *OsHsp18.0* was reversed between SH5 and Nipponbare after 6 hpi and the expression level of *OsHsp18.0* was significantly higher in SH5 than in Nipponbare throughout the rest of time points (12 to 72 hpi) (Fig. 2a). Interestingly, a sharp increase in growth of the bacteria Zhe173 on both SH5 and Nipponbare occurred between 3 to 5 dpi and the bacterial growth slowed down thereafter (Fig. 1b), which is correlated with the kinetics of *OsHsp18.0* induction.

To examine whether the induction of the *OsHsp18.0* expression is strain-specific, qRT-PCRs were performed on the RNA samples extracted from Nipponbare and SH5 leaves infected with four additional *Xoo* strains for 5 d. As shown in Fig. 2b, the expression of *OsHsp18.0* in SH5 was significantly induced by PXO79, PXO71, PXO99, and PXO280 strains (*p* < 0.01), indicating that the induction of *OsHsp18.0* expression is not in a strain-specific manner.

### OsHsp18.0 functions as a molecular chaperone *in vitro*

**Figure 3:**
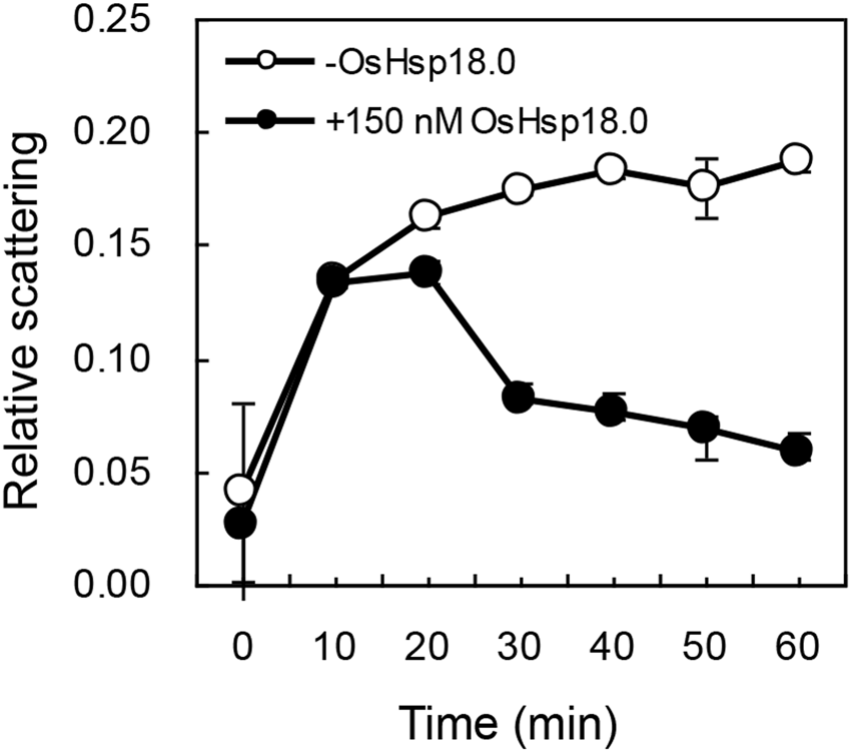
OsHsp18.0 protects LDH from thermal aggregation. 150 nM LDH monomers were incubated at 46 °C in the absence or presence of 150 nM OsHsp18.0. Relative scattering (expressed in arbitrary units) indicative of substrate aggregation was measured as the readings of absorbance at 320 nM. Error bars represent the standard deviation from at least three replicates.

Transcript level of *OsHsp18.0* in both Nipponbare and SH5 was rapidly and drastically induced by over 3000-fold within 0.5 h of heat shock treatment at 45 °C and remained significantly high (*p* < 0.01) thereafter compared at 28 °C until 3 h after heat treatment (Fig. 2c). Consistent with the result reported previously^15^, heterologous expression of OsHsp18.0 could increase thermotolerance in *E. coli* (Fig. S1). It has been reported that sHsp17.7 from pea can prevent heat-induced protein aggregation. And the chaperone activity of the sHsp17.7 can be measured by a well-established light scattering assay, in which suppression of an increase in light scattering over time can be used as a measurement of effectiveness of the chaperone activity^20^. To test whether OsHsp18.0 possesses chaperone activity, 150 nM lactate dehydrogenase (LDH) monomers, a commonly used substrate for chaperone activity assay *in vitro*, were inoculated at 46 °C in the absence or presence of 150 nM OsHsp18.0. As shown in Fig. 3, OsHsp18.0 started showing its effect on preventing the aggregation of LDH at 20 min and remained significantly effective until 60 min, confirming that OsHsp18.0 is a *bona fide* small Hsps with chaperone activity.

### Overexpression of *OsHsp18.0* enhances resistance against *Xoo* in susceptible variety Nipponbare

**Figure 4:**
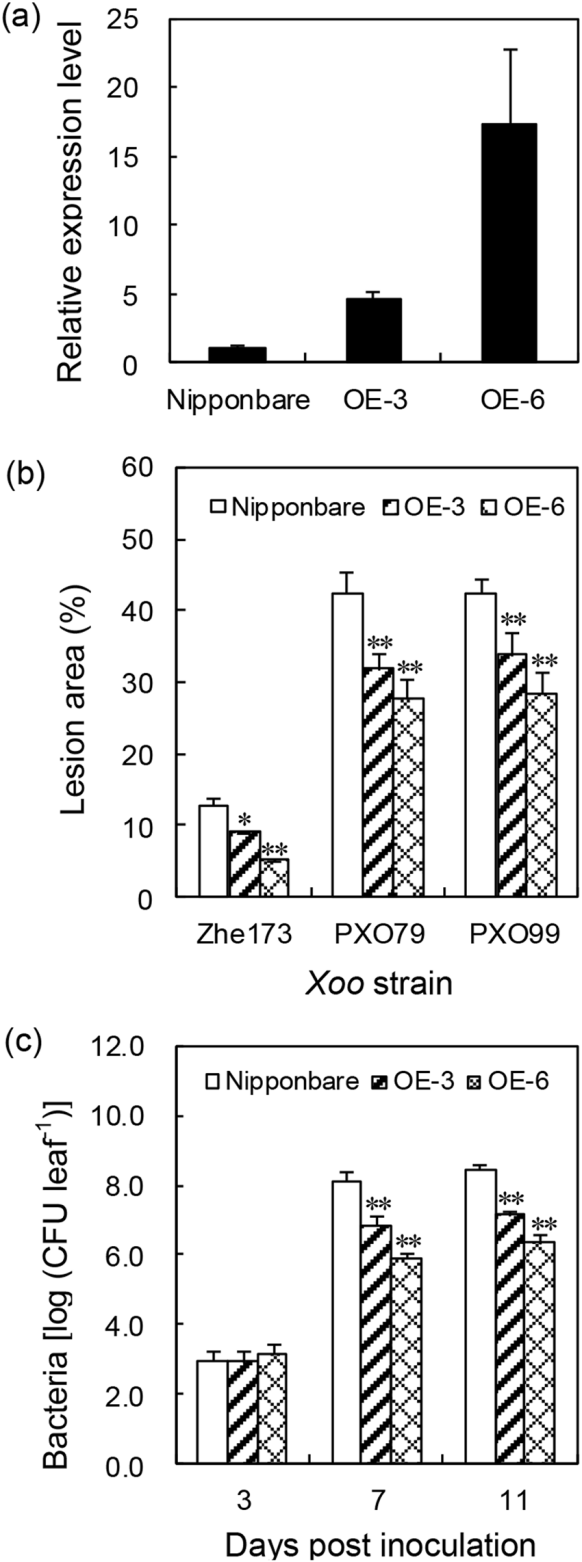
Overexpression of *OsHsp18.0* resulted in enhanced resistance to *Xoo* in a strain non-specific manner. (**a**) The relative expression of *OsHsp18.0* in transgenic lines OE-3 and OE-6 relative to that in the wild-type Nipponbare. Bars represent means (three to five replicates) ±SD. (**b**) Lesion area in wild type and transgenic lines OE-3 and OE-6 inoculated with Zhe173, PXO79 and PXO99 at 20 dpi. The asterisks indicate that a significant difference in the lesion area was detected between transgenic plants and the wild type (*p < 0.05; **p < 0.01). (**c**) Bacterial growth of Zhe173 strain in the leaves of *OsHsp18.0*-overexpressing lines OE-3, OE-6, and wild type. Bacterial CFU was counted from four leaves at each time point. The asterisks indicate that a significant difference in the CFU was detected between transgenic plants and the wild type (**p < 0.01).

To investigate the role of *OsHsp18.0* in resistance against *Xoo*, *OsHsp18.0-*overexpressing transgenic lines driven by the CaMV 35S promoter were generated in the background of Nipponbare, which is susceptible to *Xoo* (Fig. 1). qRT-PCR analysis revealed that the expression level of *OsHsp18.0* in overexpressing lines OE-3 and OE-6 was 5- and 17-fold higher, respectively, compared in Nipponbare (Fig. 4a). To test the effect of *OsHsp18.0* overexpression on the *Xoo* resistance, T_2_ progenies from lines OE-3 and OE-6 as well as wild-type plants were inoculated with strains Zhe173, PXO79 and PXO99, respectively. As shown in Fig. 4b, the lesion area on the leaves of the transgenic plants infected by these *Xoo* strains were significantly smaller than that on the leaves of Nipponbare plants. In addition, the growth rate of bacteria Zhe173 was significantly reduced at 7 and 11 dpi on the leaves of the transgenic lines OE-3 and OE-6 (*p* < 0.01) relative to on the leaves of Nipponbare (Fig. 4c). Together, these results suggest that overexpression of *OsHsp18.0* results in a broad-spectrum resistance to *Xoo* strains and OsHsp18.0 plays a positive role in basal resistance or PTI.

### Silencing of *OsHsp18.0* accelerates disease development in resistant variety SH5

**Figure 5:**
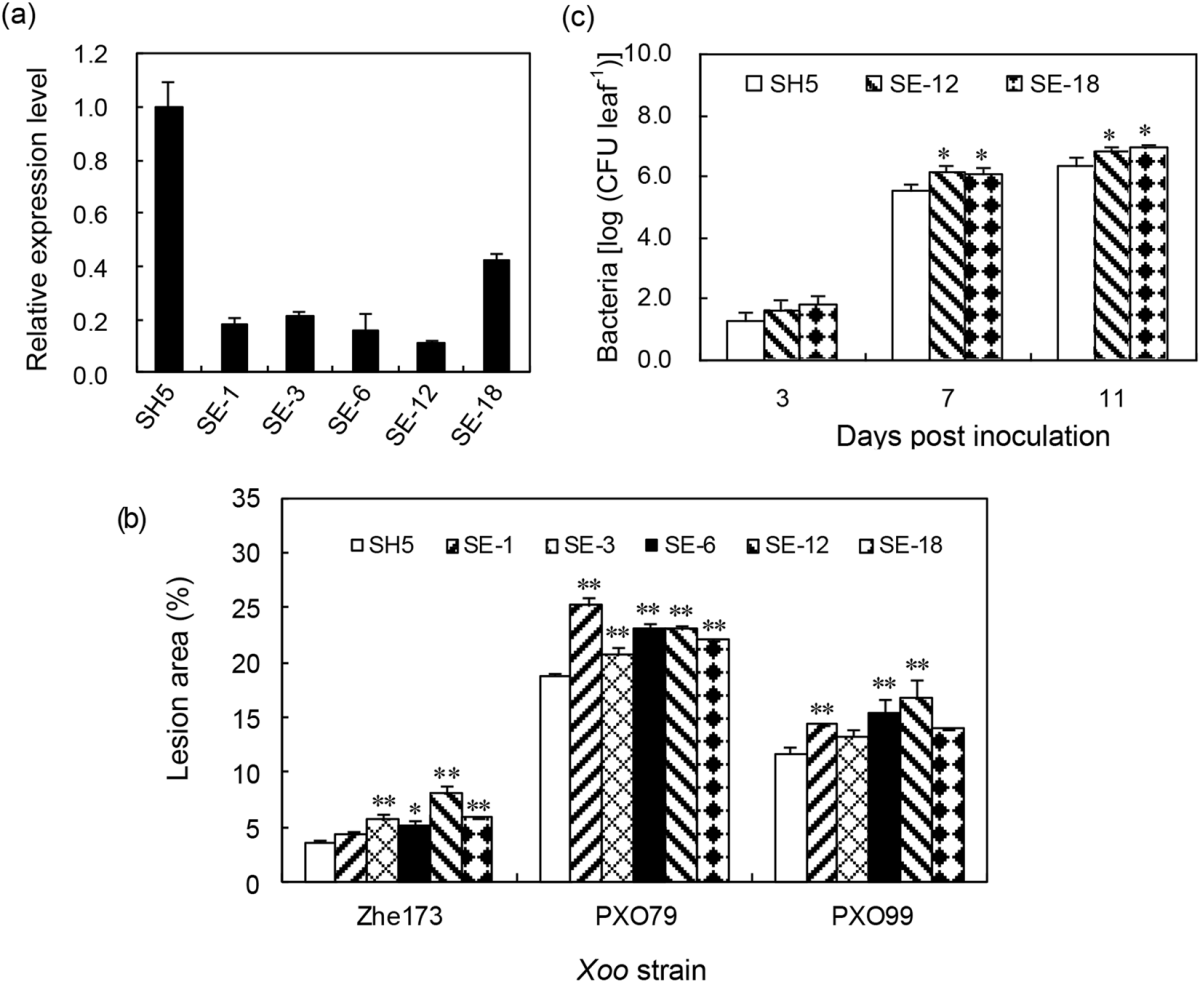
Silencing of *OsHsp18.0* increased susceptibility of SH5 to different *Xoo* strains. (**a**) Comparison of the expression levels of *OsHsp18.0* in *OsHsp18.0*-silenced transgenic plants with that in the wild-type SH5. Bars represent means (three to five replicates) ±SD. (**b**) Lesion area in wild type and T_2_ plants of five *OsHsp18.0*-RNAi transgenic lines inoculated with Zhe173, PXO79 and PXO99 at 20 dpi. The asterisks indicate that a significant difference in the lesion area was detected between transgenic plants and the wild type (*p < 0.05; **p < 0.01). (**c**) Growth rates of Zhe173 strain in the leaves of *OsHsp18.0*-silencing lines SE-12, SE-18 and wild type SH5. Bacterial CFU was counted from four leaves at each time point. The asterisks indicate that a significant difference in the CFU was detected between transgenic plants and the wild type (*p < 0.05).

To further confirm the role of *OsHsp18.0* in the resistance against *Xoo*, transgenic RNA interfering (RNAi) lines were generated in the background of SH5, which is resistant to *Xoo*. qRT-PCR analysis confirmed that the transcript level of *OsHsp18.0* was significantly silenced in five independent RNAi lines (SE-1, SE-3, SE-6, SE-12 and SE-18), where the transcript reduction varied from 60–90% (Fig. 5a). *OsHsp18.0* does not share high identity with other members of *OsHsp* family. *OsHsp19.0* (CT835445) was the mostly close related *OsHsp*, which shares only 75% identity with *OsHsp18.0*. As shown in Fig. S2, no silencing of *OsHsp19.0* was observed in the RNAi lines, indicating that the silencing in these lines was *OsHsp18.0-*specific. Next, we inoculated the T_2_ progenies of the five RNAi lines with *Xoo* strains Zhe173, PXO79 and PXO99, and the lesion area was measured at 20 dpi. As shown in Fig. 5b, the mean lesion area on the inoculated leaves of five *OsHsp18.0* transgenic lines was significantly larger than on wild-type SH5 leaves. Accordingly, the growth rate of bacteria Zhe173 was significantly increased in the lines SE-12 and SE-18 (*p* < 0.05) compared with that in SH5 (Fig. 5c), indicating that silencing of *OsHsp18.0* enhanced the *Xoo*-susceptibility in SH5 and OsHsp18.0 plays a critical role in qualitative resistance.

### *OsHsp18.0* influences the accumulation of free salicylic acid (SA)

**Figure 6:**
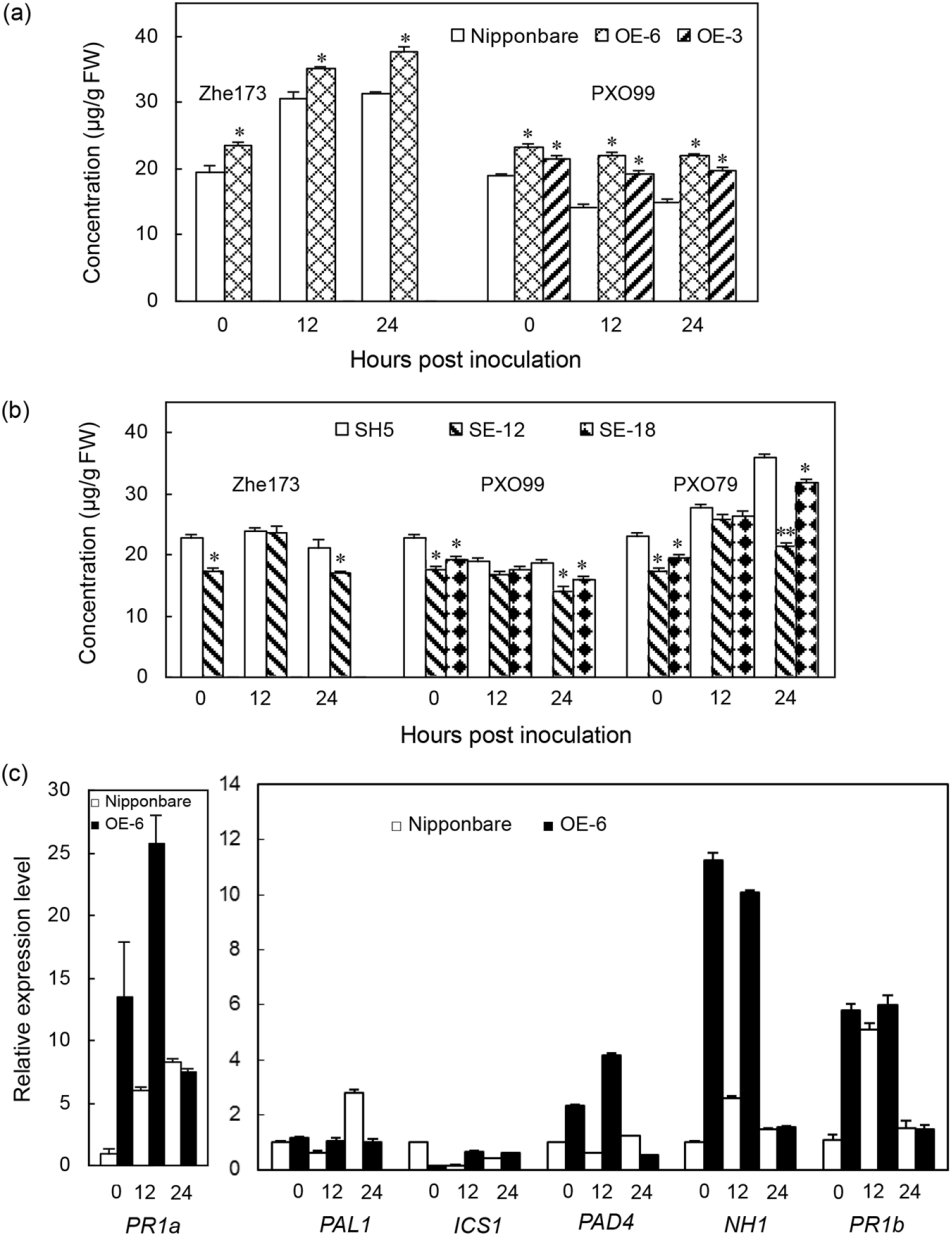
Modulating *OsHsp18.0* expression influenced the accumulation of free SA and the expression of SA-related genes. (**a**) Free SA level in the *OsHsp18.0*-overexpressing plants. (**b**) Free SA level in the *OsHsp18.0*-RNAi plants. Samples from the wild type and respective transgenic lines were collected at 0, 12 and 24 hpi with *Xoo* strains Zhe173, PXO99 and PXO79. Bars represent means (three replicates) ±SD. Asterisks indicate that a significant difference (*p < 0.05) was detected between transgenic plants and the wild type. (**c**) Effect of *OsHsp18.0* overexpressing on the expression of six SA-related genes. Samples were collected at 0, 12 and 24 hpi with *Xoo* strain Zhe173. The relative abundance of six transcripts was analyzed using qRT-PCR with primers listed in Table S1. The normalized expression level of each gene in wild-type control was set as 1. Values represent the mean ± SD from triplicate experiments.

The expression of *OsHsp18.0* in the resistant variety SH5 was induced by exogenous SA (Fig. S3). To examine whether the enhanced *Xoo* resistance observed in the *OsHsp18.0-*overexpressing lines is correlated with free SA accumulation, we quantified the endogenous free SA level in the leaves of OE-3, OE-6 and Nipponbare plants with or without subjecting to *Xoo* strain Zhe173 or PXO99 infection. As shown in Fig. 6a, Zhe173 infection induced free SA accumulation (*p* < 0.05) both in Nipponbare and OE-6 plants, but the degree of induction was greater in OE-6 than in Nipponbare. Interestingly, instead of induced in response to the PXO99 infection, the free SA level in Nipponbare was significantly reduced at 12 and 24 hpi, respectively (Fig. 6a). Contrary to what have observed in Nipponbare, the free SA level was not induced in OE-6 and OE-3 by PXO99 at 12 and 24 hpi. Although SA accumulation responded differently to different *Xoo* strains, the free SA level in *OsHsp18.0-*overexpressing plants was significantly increased (*p* < 0.05) compared with Nipponbare both before and after the infections by different *Xoo* strains (Fig. 6a). Together, these results suggest that the increased level of free SA in *OsHsp18.0-*overexpressing plants may be responsible for the enhanced resistance to *Xoo* (Fig. 4). In contrast, the free SA level in *OsHsp18.0-*RNAi plants was significantly reduced (*p* < 0.05) compared with the wild-type SH5 either in the absence of *Xoo* inoculation or at 24 hpi of Zhe173, PXO99 or PXO79 infection (Fig. 6b), further confirming that the free SA level is highly correlated with resistance to *Xoo* (Fig. 5b and c).

To test the involvement of SA in disease resistance in rice, we analyzed the expression of six genes either associated with SA biosynthesis or activation of the SA-dependent pathway in response to Zhe173 attack by qRT-PCR. Phenylalanine ammonia lyase 1 (PAL1; X16099) is involved in SA biosynthesis through the phenylpropanoid pathway. Isochorismate synthase 1 (ICS1; AK120689) and phytoalexin-deficient 4 (PAD4; CX118864) are putatively involved in SA biosynthesis in rice by the isochorismate pathway^17^. Induced expression of acidic pathogenesis-related protein 1 (PR1a; AJ278436) and *Arabidopsis* NPR1 ortholog (NH1; AY923983), are associated with activation of the SA-dependent pathway^21^. Basic PR protein 1 (PR1b; U89895) appears to function in both JA- and SA-dependent pathways^17, 21^. As shown in Fig. 6c, the expression of *PAL1*, *PAD4*, *PR1a*, *NH1* and *PR1b* genes were significantly increased (*p* < 0.01) in the OE-6 line compared with Nipponbare in at least one time points examined. Especially, *OsHsp18.0-*overexpressing plants, which acquired enhanced resistance to *Xoo*, showed markedly increased expression of two activation-related genes of SA-dependent pathway, *PR1a* and *NH1*, when without and with pathogen infection. These results indicate that *OsHsp18.0* positively regulate *Xoo*-resistance may be dependent on SA.

### Constitutive expression of *OsHsp18.0* in rice confers tolerance to both heat and salt stress

**Figure 7:**
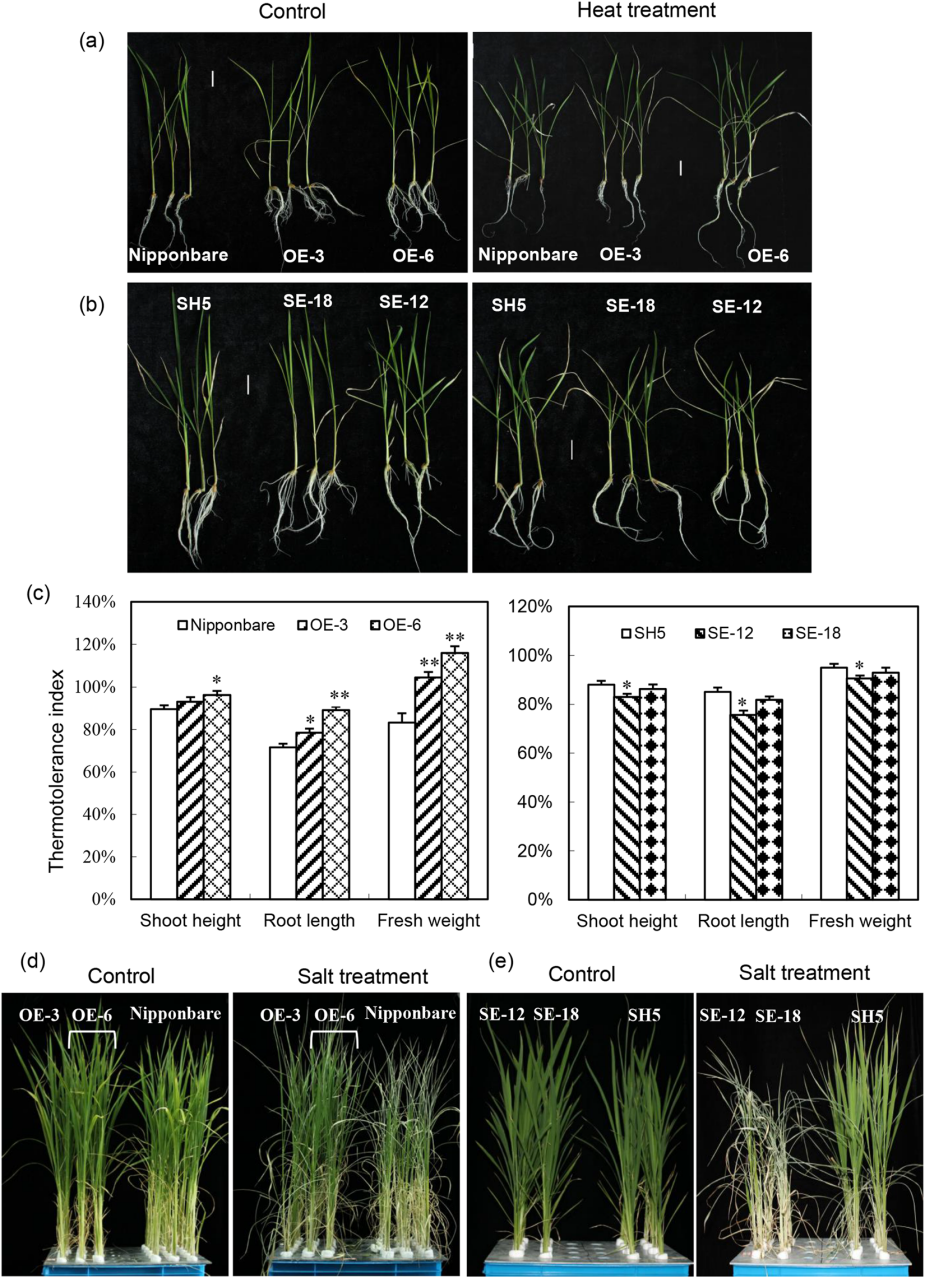
The roles of OsHsp18.0 in thermo- and salt-tolerance. (**a**) *OsHsp18.0*-overexpressing lines OE-3 and OE-6 exhibited higher thermotolerance than wild-type Nipponbare. The 4-d seedlings of Nipponbare and transgenic lines were subjected to heat treatment. The representative plants were photographed after recovery at 28 °C for 14 d. Bars = 2 cm. (**b**) The thermotolerance of *OsHsp18.0*-silenced transgenic plants SE-18 and SE-12 was reduced compared with wild-type SH5 plants. (**c**) Comparisons of the thermotolerance indexes between transgenic and wild-type plants. The thermotolerance indexes were calculated as described in Materials and Methods. The asterisks indicate that a significant difference (*p < 0.05; **p < 0.01) was detected between transgenic plants and the wild type. (**d**) *OsHsp18.0*-overexpressing transgenic lines OE-3 (column 1) and OE-6 (column 2–3) displayed enhanced tolerance to salt stress relative to the wild-type Nipponbare. (**e**) *OsHsp18.0*–silencing lines SE-18 and SE-12 were more sensitive to 200 mM NaCl than wild-type SH5. The photos in (**d**) and (**e**) were taken at 5 d post salt treatment.

It has been shown previously that overexpression of CI sHsps enhances thermo- and salt-tolerance in a number of plant species^3, 22–24^. To examine whether the OsHsp18.0 has the same effects on abiotic stresses, the 4-d seedlings of Nipponbare, *OsHsp18.0*-overexpressing lines, together with SH5 and *OsHsp18.0-*RNAi lines were subjected to the following heat treatment regime as described previously^25^: 42 °C for 2 h; followed by recovery at 28 °C for 2 d; then heat shock at 47 °C for 70 min again, finally at 28 °C for 14 d. Consistent with previous reports, the seedlings of *OsHsp18.0-*overexpressing lines OE-3 and OE-6 exhibited enhanced thermotolerance compared with wild-type Nipponbare (Fig. 7a). The thermotolerance index (FW of heat-treated seedlings/FW of control seedlings) of OE-3 and OE-6 was increased by 25% and 39%, respectively, compared with that of Nipponbare (Fig. 7c). In contrast, the seedlings of *OsHsp18.0*-RNAi lines SE-18 and SE-12 displayed enhanced sensitivity to heat treatment compared with SH5 (Fig. 7b). The thermotolerance index of SE-18 and SE-12 was decreased by 2.1% and 4.6%, respectively, relative to SH5 (Fig. 7c). The thermotolerance indexes calculated based on shoot height or root length shared a similar trend with the FW both in overexpressing and RNAi lines in response to heat treatment (Fig. 7c).

As the expression of *OsHsp18.0* was salinity-inducible^13^ (Fig. S3), we then examined whether the OsHsp18.0 plays a role in salinity tolerance. To achieve this, five-leaf stage plants of transgenic overexpressing and RNAi lines of *OsHsp18.0* together with their respective wild-type plants (Nipponbare or SH5) were subjected to 200 mM NaCl treatment for up to 5 d. While the seedlings of *OsHsp18.0-*overexpressing lines OE-3 and OE-6 exhibited enhanced tolerance to 200 mM NaCl relative to Nipponbare (Fig. 7d), the *OsHsp18.0*-RNAi lines SE12 and SE18 displayed significantly enhanced sensitivity compared with SH5 (Fig. 7e). The *OsHsp18.0-*silenced plants almost dried out for the 5-d treatment, whereas most leaves of the wild-type SH5 plants were still green (Fig. 7e). Together, these results strongly suggest that OsHsp18.0 functions as a positive regulator in rice tolerance to both high temperature and salinity.

### OsHsp18.0 is a cytoplasm- and nuclear envelope-localized protein

**Figure 8:**
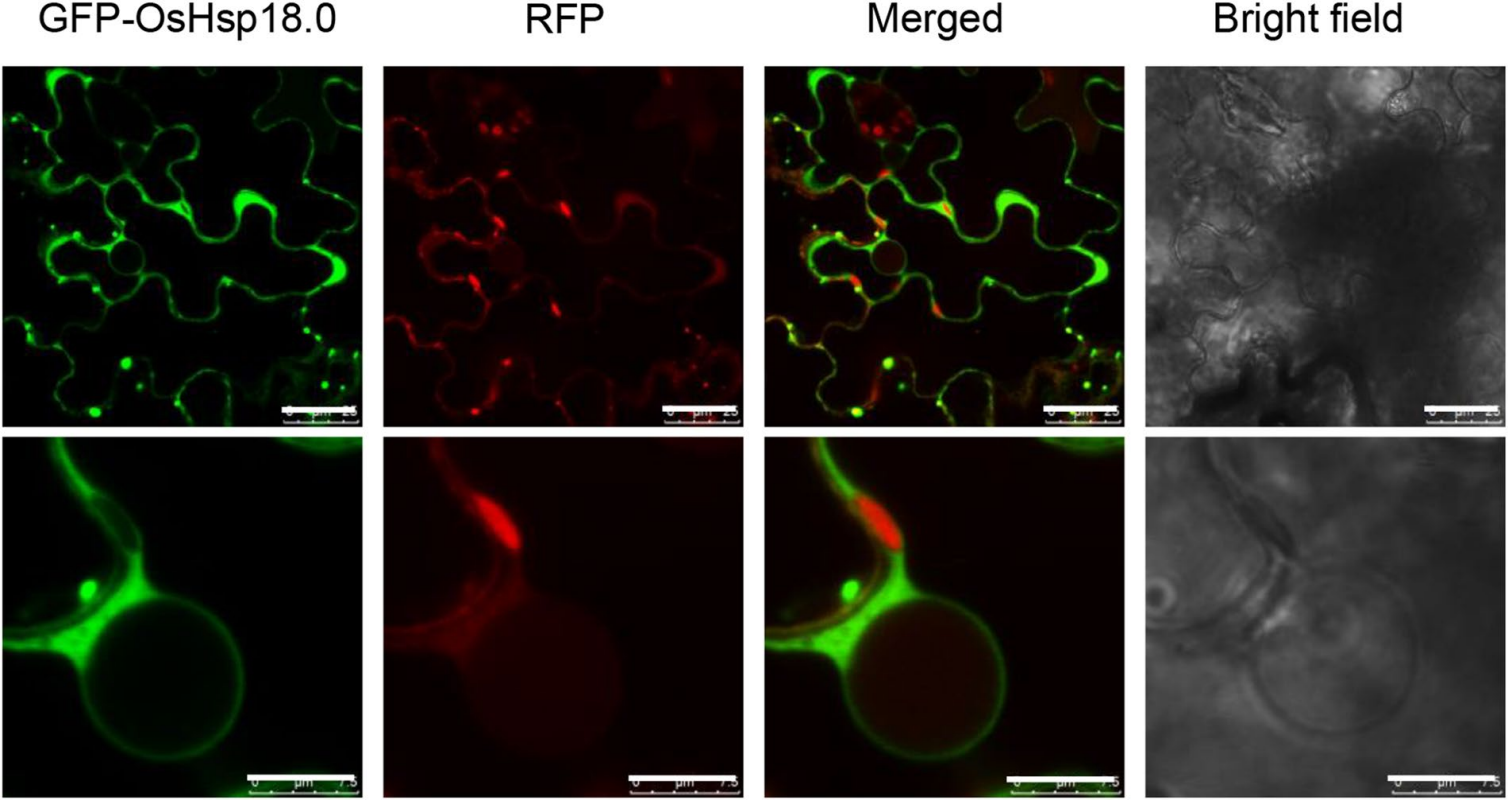
OsHsp18.0 is localized in both cytoplasm and nuclear envelope. *Agrobacteria* solutions carrying GFP-OsHsp18.0 and free RED constructs driven by the 35S promoter were infiltrated into *N. benthamiana* leaves, respectively. At 2 d post infiltration, the infiltrated leaf areas were cut and the fluorescence images were captured by confocal laser scanning microscopy. The white bar represents 25 μm (top row) or 7.5 μm (bottom row).

In addition to the conserved ACD domain, a nuclear localization signal (NLS) KKPK was predicted at amino acids 156–159 of OsHsp18.0 using the PSORT program (http://psort.hgc.jp/form.html). To investigate the subcellular localization of OsHsp18.0, its coding sequence was fused with green fluorescent protein (GFP) to generate a 35S-GFP-OsHsp18.0 fusion construct, and the resulting plasmid was transiently overexpressed in *Nicotiana benthamiana* leaf cells. As shown in Fig. 8, GFP-OsHsp18.0 was predominantly localized in the cytoplasm and was particularly enriched on the nuclear envelope, but no GFP signal was detected inside the nucleus, suggesting that OsHsp18.0 functions in the cytosol and on the nuclear envelope. As a control, free red fluorescent protein (RFP) was coexpressed and RFP signal was present both in the cytoplasm and nucleus (Fig. 8).

## Discussion

sHsps fulfill their task as molecular chaperones by stabilizing early unfolding intermediates of aggregation-prone proteins, arising as a result of diverse stress conditions. Under cellular stress conditions, sHsps selectively bind nonnative proteins, prevent their aggregation, and maintain them in a competent state for subsequent ATP-dependent folding by Hsp70 s and Hsp100 s and its cochaperones^6^. Unlike other chaperones, sHsps do not require ATP to bind substrate proteins, and they have a very high capacity for binding denatured substrates^5, 6, 9, 11^. In mammalian cells and plants, the Hsp70/Hsp40 system is required for refolding of substrate proteins bound to sHsps suggesting that sHsps collaborate with Hsp70/Hsp40 in a relay to fulfill its function. Supporting this, it has been shown that both Hsp70 s and its cochaperone Hsp40 are critical for basal resistance in *Arabidopsis* and soybean, respectively^26, 27^. It was predicted that simultaneously modifying the expression of *sHsps*, *Hsp70* and *Hsp40* will have a great chance to improve the resistance/tolerance of plants against diverse stresses significantly.

Previous studies have demonstrated that a few members of *sHsps* from different plant species have chaperone activity *in vitro* or *in vivo* under heat treatment^1, 2, 5, 20, 28–30^. Heterologous expression of some plant *sHsps* in *E. coli* increases its thermo- and cold-tolerance and the protective effects of sHsps are associated with increased stability of soluble proteins^15, 31, 32^. In addition, overexpressing plant *sHsps* also confers resistance to heat, cold and salt^1, 33^. Similarly, we showed that the OsHsp18 can function as a molecular chaperone in increasing the thermotolerance of *E. coli* cells *in vivo* (Fig. S1) and preventing thermal aggregation of a client protein (Fig. 3); overexpressing *OsHsp18.0* in rice not only increased the tolerance to heat and salt stresses (Fig. 7), but also significantly enhanced the resistance against *Xoo* (Fig. 4), assigning a new function for sHsps in rice.

The involvement of sHsps in disease resistance has been shown by loss-of-function studies previously. Silencing *Ntshsp17* in *N. benthamiana* compromises resistance to both pathogenic (PTI) and non-pathogenic *Ralstonia solanacearum* (ETI) in a hypersensitive response (HR)-independent manner^9^ and silencing of a gene encoding a CI type sHsp20, RSI2 (Required for Stability of I-2), which interacts with the LRR domain of the tomato R protein I-2 in *N. benthamiana*, compromised the HR that is normally induced by auto-active variants of I-2^34^, indicating that sHsps play critical roles in PTI, ETI and HR induction. Here, we confirmed the positive roles of rice OsHsp18.0 in disease resistance by both gain-of-function and loss-of-function approaches (Figs 4 and 5). Overexpressing *OsHsp18.0* conferred the enhanced *Xoo* resistance to a susceptible variety (Fig. 4), and silencing *OsHsp18.0* compromised the *Xoo* resistance in a resistant variety (Fig. 5). The fact that SH5 exhibits broad spectrum resistance to 11 different *Xoo* strains^35^, suggests that the resistance conferred by SH5 is not an R protein-mediated resistance, as it is not race-specific (Figs 2 and 5). Even though both SA synthesis and pathway-activated gene expressions were significantly induced in the *OsHsp18.0-*overexpressing line in response to *Xoo* infection, no HR was visible on these plants (Fig. S4). Consistent with this, transient overexpression of *OsHsp18.0* in *N. benthamiana* leaves also did not trigger HR (Fig. S5). These results further confirm that the *Xoo* resistance presenting in SH5 is independent of R proteins. This is not surprising given that the molecular mechanisms of rice qualitative resistance to *Xoo* are largely different from those of NB-LRR type R-protein-mediated resistance in other plant pathogen pathosystems^18, 36^. Although the rice genome encodes over 600 NB-LRR genes, only one (*Xa1*) of the seven cloned major resistance genes against *Xoo* encodes an R protein^19^. Instead, transcription activator-like effector-mediated transcriptional activation or suppression of major resistance genes plays an important role in rice qualitative resistance to *Xoo* ^18^.

Emerging evidence showed that SA plays a role in rice basal defense^37^. The free SA level was not always induced by any *Xoo* strains, but significantly increased in *OsHsp18.0-*overexpressing lines and decreased in *OsHsp18.0-*RNAi lines compared in respective wild-type plants (Fig. 6a and b), suggesting that overexpressing *OsHsp18.0* sensitizes the SA-dependent resistance and OsHsp18.0–mediated *Xoo* resistance does require the activation of SA signaling. As HRs were not involved in the *Xoo* resistance in the *OsHsp18.0-*overexpressing lines (Fig. S4), the growth and development of these lines were not affected under non-stress conditions (Fig. S6). In addition, both the *Xoo* resistance and abiotic tolerance of these lines were simultaneously induced only under stress conditions (Figs 4 and 7), suggesting a general role of OsHsp18.0 in stress tolerance and disease resistance. Unlike R protein mediated resistance, which is often associated with a HR, this type of induced resistance/tolerance is particularly important for crop improvement as it confers a broad spectrum resistance/tolerance to both biotic and abiotic stresses simultaneously without compromising normal growth and development.

The non-specific induction of *OsHsp18.0* by both abiotic and biotic stresses (Figs 2 and S3)^4, 13^ and the non-specific binding of OsHsp18.0 to its client proteins in a substrate nonspecific manner (Fig. 3) strongly suggests that OsHsp18.0 most likely plays a general role under both abiotic and biotic stress conditions. This statement is supported by the fact that OsHsp18.0 plays positive roles in both *Xoo* resistance and heat/salt tolerance (Figs 4, 5 and 7). It is possible that OsHsp18.0 may act as a molecular chaperone and nonspecifically prevent the aggregation/misfolding of intracellular proteins that are positively involved in *Xoo* resistance, as well as in heat and salt tolerance^8^.

The presence of strong GFP-OsHsp18.0 on the nuclear rim (Fig. 8) suggests its potential function on the nuclear envelope (NE), which is the double membrane surrounding the eukaryotic cell nucleus. It has been shown that nucleo-cytoplasmic trafficking of proteins and RNAs is mediated through nuclear pore complexes (NPCs). NPCs are large protein complexes that cross the nuclear envelope and each NPC contains approximately 30 constituent nucleoporin proteins (Nups)^38^. Impairments in cytoplasmic protein import as well as nuclear protein and mRNA exports, due to the mutations in genes encoding Nups, compromise both biotic and abiotic stress responses^39^. Partial loss-of-function of MOS7, an integral NPC homologous to Nup88 results in reduced nuclear accumulation of the important defense regulators EDS1, NPR1 and snc1 and thus compromised PTI, ETI and systemic acquired resistance^40^. Disruption of components required for nucleo-cytoplasmic trafficking results in defects in basal and R protein mediated immunity^40, 41^ as well as in cold-, drought- and thermo-tolerance^42–44^. Overexpression of *OsHsp18.0* enhanced both the basal resistance to *Xoo* (Fig. 4) and heat/salt tolerance (Fig. 7), while silencing *OsHsp18.0* reduced both the qualitative resistance to *Xoo* (Fig. 5) and heat/salt tolerance (Fig. 7), suggesting that OsHsp18.0 plays broad roles both in biotic and abiotic responses. It is possible that OsHsp18.0 is required for preventing misfolding or aggregation and thus maintaining the proper conformations of the proteins involved in nucleo-cytoplasmic trafficking under stress conditions. This could partially explain the correlation between the enhanced transcript levels of *OsHsp18.0* in resistant cultivar than in susceptible cultivar in response to *Xoo* infection (Figs 1 and 2).

## Materials and Methods

### Plant materials and abiotic stress treatments

SH5 is a stable somatic hybrid between *japonica* var 8411 and the wild rice *Oryza meyeriana* Baill that exhibits a broad spectrum of resistance to *Xoo* strains at the seedling, tillering and booting stages^35, 45^. *Japonica* var Nipponbare is a susceptible variety to *Xoo* strains^21^. Rice seedlings were grown hydroponically in a growth chamber under 16 h light/8 h dark photoperiod (350–400 μmol/m^2^/s) at 28 °C during the daytime and 25 °C at night, respectively. Five-leaf stage seedlings were exposed to heat (45 °C) or high salinity (200 mM NaCl). *N. benthamiana* plants were grown in a growth chamber at 25 °C during the daytime and 23 °C at night, respectively, with a photoperiod of 16 h light/8 h dark.

### *Xoo* inoculation and disease evaluation

A strongly virulent *Xoo* strain Chinese pathotype IV (Zhe173), predominant in Yangtze River valley in China^45^, as well as four Philippine strains (PXO79, PXO71, PXO99 and PXO280) were used in this study. Bacterial inoculum was prepared from 48 h culture on potato sucrose agar slants and its density was adjusted to 10^9^ colony-forming units (CFU)/mL. The fully expanded uppermost leaves at five-leaf stage were inoculated by the leaf-clipping method^17^. The inoculated leaves were harvested at the indicated time point, and bacterial growth in leaves was monitored by plate counting CFU^46^. The disease was also evaluated by measuring the lesion length at 14–20 dpi. Lesion area was calculated as a percentage of lesion length relative to leaf length^16^.

### Cloning of the full-length cDNA

Total RNA was extracted from leaves with TRIzol reagent and treated with DNase I (RNase free; Takara Shuzo, Kyoto, Japan). First-strand cDNA was synthesized using M-MLV reverse transcriptase (Promega, Madison, WI, USA) and oligo-dT primer. The specific primers of *OsHsp18.0* (5′-TCGAGAAGCCACAAACCC-3′ and 5′-CGCATACGGCATACAGACC-3′) were designed based on the sequence of GenBank accession number AK071240. The PCR was conducted for 35 cycles each consisting of 30 s at 95 °C, 30 s at 52 °C and 1 min at 72 °C. Purified amplified products were ligated to the pMD18-T vector for sequencing (Sangon Biotech, Shanghai, China).

### Gene expression analysis by qRT-PCR

The specific primers of *OsHsp18.0* (5′-AGGAGGAGAGGCTGCTGGTGAT-3′ and 5′-CGATGGTCTTGGGCTTCTTGGG-3′) were designed to amplify 237 bp of fragment, which were tested for their specificity by reverse transcription-PCR and also by qRT-PCR, followed by agarose gel electrophoresis and melting curve analysis, respectively. The primers used for qRT**-**PCR of SA-related genes were listed in Table S1. The reaction was performed in quadruple replicates for each sample in 20 μL final volume containing 2 μL diluted cDNA, 10 pmol of each primers, 0.4 μL 50 × ROX and 10 μL of SYBR qPCR Mix (Toyobo, Osaka, Japan). PCR was run in a StepOnePlus (Applied Biosystems, Foster City, CA, USA) using the following cycling regime: 95 °C for 1 min, followed by 35 cycles of 95 °C for 5 s and 60 °C for 30 s. The relative expression levels of the amplified products were calculated based on the comparative threshold cycle (C_T_) method. Transcript abundance was normalized against the reference gene *β*-actin (X15865).

### Heterologous expression and purification of OsHsp18.0 in *E. coli*

The coding region of *OsHsp18.0* (DQ180746) was amplified using the primers 5′-CGGAATTCATGGAGAGCGCCATGTTCGGGCTGG-3′ and 5′-CCCTCGAGTCACGCGACCTTGACCTCGCTGGTC-3′, which contain *Eco*RI and *Xho*I restriction enzyme sites (underlined), respectively. The PCR product was double digested with *Eco*RI and *Xho*I and ligated into a pGEX vector that was modified to express a 9× -histidine peptide fused to the N-terminus of GST^47^. After sequence confirmation, the resulting pGEX-*OsHsp18.0* recombinant vector was introduced into *E. coli* strain BL21 (DE3; Novagen, Madison, WI, USA).

Protein expression and purification were performed as described previously^48^. Briefly, isopropyl *β*-D-thiogalactopyranoside was added to the cells to induce the expression of OsHsp18.0. The soluble cell fraction was separated by a Ni^2+^-NTA His·Bind Sepharose Superflow (Novagen, Darmstadt, Germany) and a Glutathione-Sepharose^TM^ 4 Fast Flow column (GE Healthcare Life Sciences, Uppsala, Sweden). Next, the affinity tags were proteolytically clipped from the fusion protein by use of PreScission Protease (Sangon Biotech, Shanghai, China). The tags and protease were removed by passing the proteolysate through a Glutathione-Sepharose^TM^ 4 Fast Flow. The purified protein was examined by SDS-PAGE and the aliquots were stored in −80 °C and used for subsequent assays.

### Thermal aggregation experiments

Aggregation protection of LDH from rabbit muscle (Roche Applied Science, Pleasanton, CA, USA) was assessed using the method as follows^20^: 150 nM LDH was combined with 150 nM of purified OsHsp18.0 in 50 mM HEPES-KOH, pH 7.5. Samples were incubated in 1 mL quartz cuvettes in a thermostated water bath at 46 °C. To quantify the changes in light scattering, absorbance reading at 320 nM in a UV–visible spectrophotometer (Cary 4000, Agilent Technologies, Australia) was taken at 0, 10, 20, 30, 40, 50 and 60 min post heating treatment.

### Vector construction and rice transformation

*OsHsp18.0* cDNA was amplified with specific primers 5′-CGGGGTACCATGGAGAGCGCCATGTTC-3′ and 5′-CTAGTCTAGAGGAATCTCATCACGCGAC-3′, which contain *Kpn*I and *Xba*I restriction sites (underlined), respectively. The PCR product was confirmed, and double digested by *Kpn*I and *Xba*I, before ligating into the binary vector pCAMBIA1301 driven by the 35S promoter of CaMV. For the RNAi construct, 319 bp of *OsHsp18.0* ORF (Fig. S2) in sense and antisense orientation was constructed into both sides of the second intron of the maize *NIR1* gene. The fragment was then cloned into pCAMBIA1301. The recombinant plasmids were introduced into *Agrobacterium tumefaciens* strain EHA105 by the freeze-thaw method. The overexpression vector was used to transform into rice var Nipponbare, and the RNAi vector was transformed into SH5. *Agrobacterium*-mediated transformation was performed using calli derived from mature embryos. Transformed calli were selected for hygromycin resistance, and the transgenic plants were regenerated subsequently. PCR screening of T_0_, T_1_ and T_2_ plants was carried out using primers specific for the *OsHsp18.0* cDNA to select homozygous lines.

### Quantification of SA

The SA samples were prepared and extracted with slight modification of the procedure of Marek *et al*.^49^. In brief, three replicates of each ground sample weighing ~1000 mg were placed in 1 ml of 80% methanol. The organic extracts containing free SA were quantified using the Agilent 1260 HPLC system with a Zorbax SB-C18 column (Agilent Technologies, Palo Alto, CA). Separations were performed using a gradient of increasing methanol content. SA was detected at 296 nM excitation and 410 nM emission by using fluorescence detector^50^.

### Stress treatments of transgenic rice plants

Seeds of four lines of T_2_ generation transgenic rice were soaked in water for 1 d, and germinated seeds were shifted to agar medium containing 25 mg/mL hygromycin for 4 d. The seedlings were transferred to nutrient solution at 28 °C normal conditions. For the heat stress experiment, 4-d seedlings were shifted at different temperatures as following regime^25^: 42 °C for 2 h −28 °C for 2 d −47 °C for 70 min and −28 °C for 14 d. The FW of whole plant, shoot height and root length were measured, respectively, and the thermotolerance index was calculated (heat-treated/control). For salt stress treatment, five-leaf seedlings were shifted to nutrient solution containing 200 mM NaCl for 5 d.

### Subcellular localization of GFP-OsHsp18.0

GFP under the control of the 35S promoter of CaMV from pAVA321 vector was cloned into pCAMBIA1300 vector as a GFP control and named pCAMBIA1300-35S-GFP. To generate GFP fusion proteins, the full-length cDNA of *OsHsp18.0* gene was amplified by PCR using the forward (5′-CGCGGATCCGAGAGCGCCATGTTC-3′) and the reverse (5′-TGCTCTAGACATCACGCGACCTTGAC-3′) primers for GFP fusion, where underlined nucleotides indicate *Bam*HI and *Xba*I restriction sites, respectively. PCR products were inserted into the PMD-18T vector. Validated cDNA inserts were then subcloned into binary vector pCAMBIA1300-35S-GFP by double digestion with *Bam*HI and *Xba*I. The resulting fusion construct (35S-GFP-*OsHsp18.0*) and the empty vector (35S-GFP) were transferred into *A. tumefaciens* strain GV3101, while empty vector 35S-RFP into *A. tumefaciens* strain GV2260. Transient expression of fusion proteins in *N. benthamiana* leaves via agroinfiltration as described previously^51^. Images were captured using confocal laser scanning microscopy (Leica TCS SP5 AOBS, Wetzla, Germany). Excitation wavelengths were 488 nM for GFP and 550 nM for RFP. Emission was detected at 505 to 530 nM for GFP and 570 to 610 nM for RFP.

### Statistical analysis

The experiments were repeated at least three times and SD was calculated. Comparison of two treatment groups was performed using a Student’s *t*-test. A one-way analysis of variance (ANOVA) was performed on groups of plants that had more than two groups. Means that were significantly different were compared post-hoc using Tukey’s *t*-tests. Data points representing statistically significant differences are marked with asterisk (*p < 0.05; **p < 0.01).

## Electronic supplementary material

SUPPORTING INFORMATION
