# Supplementary material for: A Class II small heat shock protein OsHsp18.0 plays positive roles in both biotic and abiotic defense responses in rice
Source: Sci Rep. 2017 Sep 12;7:11333. doi: 10.1038/s41598-017-11882-x (PMC5595972; doi:10.1038/s41598-017-11882-x)
Supplement: Supplementary file 1 — SUPPORTING INFORMATION [file 41598_2017_11882_MOESM1_ESM.doc]

**Title page**

**The full title:** A Class II small heat shock protein OsHsp18.0 plays positive roles in both biotic and abiotic defense responses in rice

**Author list:** Jie Kuang^1,†^, Jianzhong Liu^1, †^, Jun Mei^1, †^, Changchun Wang^1^, Haitao Hu^1^, Yanjun Zhang^1^, Meihao Sun^1^, Xi Ning^1^, Langtao Xiao^2^ & Ling Yang^1,*^

^1^College of Chemistry and Life Sciences, Zhejiang Normal University, Jinhua, Zhejiang, 321004 China

^2^Hunan Provincial Key Laboratory of Phytohormones and Growth Development, Hunan Agricultural University, Changsha, Hunan, 410128 China

^†^These authors contributed equally to this work.

^*^Corresponding author: E-mail: yangl@zjnu.edu.cn

**SUPPORTING INFORMATION**

**Table S1.** Specific primers of pathogenesis-related genes used for qRT-PCR.

Name Accession No. Forward primer ( 5’-3’) Reverse primer ( 5’-3’) Length

*PR1a* AJ278436 TCGTATGCTATGCTACGTGTTT CACTAAGCAAATACGGCTGACA 154 bp
*PR1b* U89895 GGCAACTTCGTCGGACAGA CCGTGGACCTGTTTACATTTTC 118 bp
*ICS1* AK120689 TATGGTGCTATCCGCTTCGAT CGAGAACCGAGCTCTCTTCAA 120 bp
*NH1* AY923983 CACGCCTAAGCCTCGGATTA TCAGTGAGCAGCATCCTGACTAG 120 bp
*PAD4* CX118864 GCCAGCTCCCCTACGACTTC CGTGTGCGGTGTAGGTTGTT 120 bp
*PAL1* X16099 GGGCAACCCAGTGACCAA CGATTGCCTCGTCGGTCTT 100 bp
*Hsp19.0* CT835445 GGTGAAGTACCTGAGGATGGA TACTTGCCCTTGCCCTTGG 213 bp
*β*-actin X15865 TTGCCAAGGCTGAGTACGACGA AAACAAGCAGGAGGACGGCGAT 120 bp

**Figure S1.** Thermotolerance of the *E. Coli* cells expressing *OsHsp18.0* fusion protein. The cells carrying pSKB.4 and *OsHsp18.0*-pSKB.4 were incubated at 37 °C until they reached the midlog phase (OD_600_ = 0.6). The concentration was diluted 10-fold in three series. One mL of each sample was spotted onto the Luria-Bertani plates with 0.5 mM IPTG induction, and then the plates were subjected to 37 °C, 50 °C and 70 °C treatments for 30 min, respectively. After overnight at 37 °C, the plates were photographed. The experiment was repeated three times with similar result.

37 °C

50 °C

70 °C


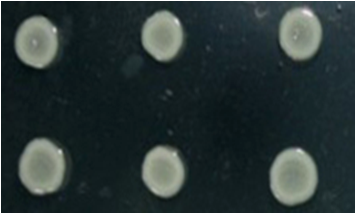

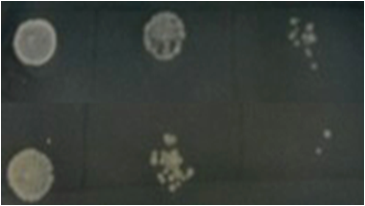

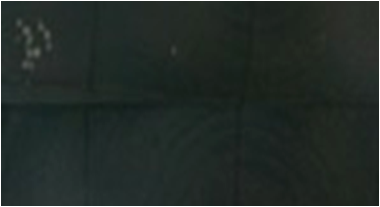


*OsHsp18.0*-pSKB.4

pSKB.4

**Figure S2.** (a) Multiple alignment of the nucleic acid sequences of *OsHsp18.0* used for RNAi construct with the corresponding sequences of the four most closely related members. Identical or different residues are highlighted by dark or gray shading, respectively. (b) The expression level of the *OsHsp19.0* in the leaves of *OsHsp18.0* RNAi transgenic lines SE-12 and SE-18. The expression level was normalized using *β*-actin as an internal reference, and set the expression in the SH5 leaves as 1. Bars represent means (three replicates) ± SD.


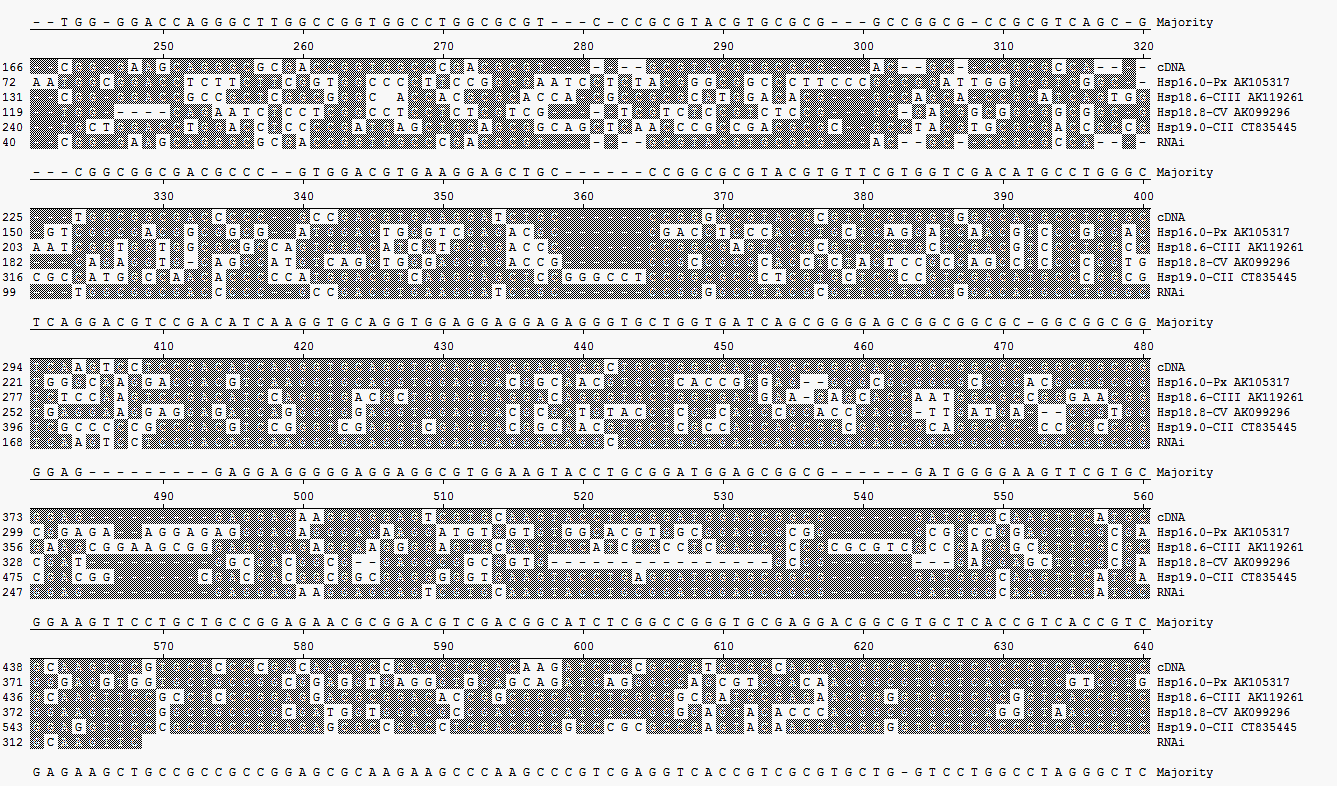


(a)

(b)

Relative expression level


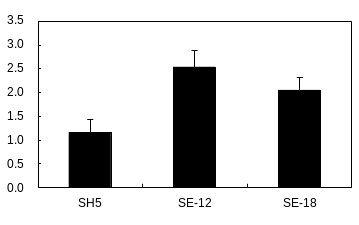


**Figure S3.** The relative expression of *OsHsp18.0* in rice leaves of SH5 after treated with 2 mM SA (a), or 200 mM NaCl (b). The expression level was normalized using *β*-actin as an internal reference, and set the expression in the SH5 leaves at 2 h as 1. Bars represent means (three replicates) ± SD. The asterisks indicate that a significant difference in expression was detected between treated plants and the control group (**, *P*<0.01).

****

****

****

Hours after treatment

Relative expression level

(a)

Hours after treatment

(b)

Relative expression level

****

****

****

****

****

****

**Figure S4.** Lesion development in wild-type Nipponbare and the plants of two *OsHsp18.0*-overexpressing transgenic lines at 20 dpi infected with *Xoo* strain Zhe173.

Wild-type Nipponbare

OE-3

OE-6

OE-6


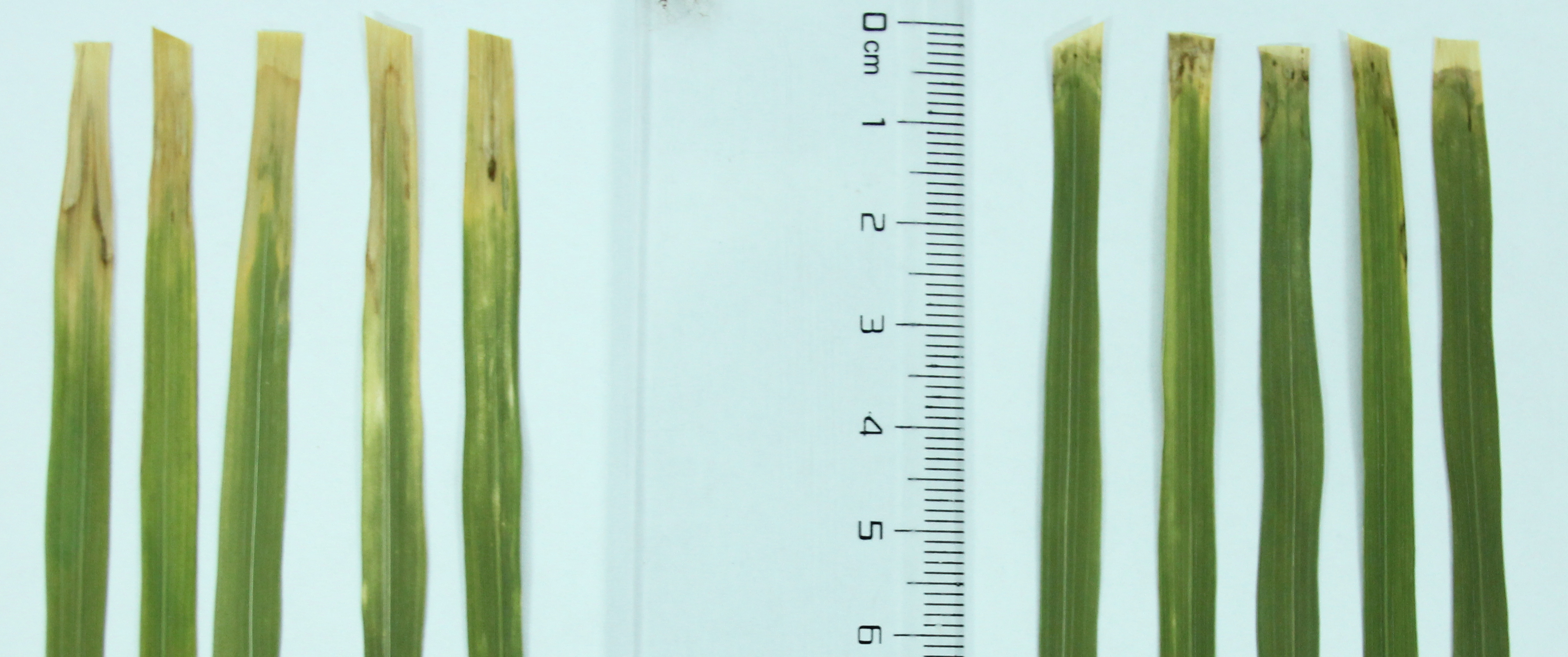


**Figure S5.** Transient overexpression of *OsHsp18.0* in *N. benthamiana* leaves did not cause hypersensitive response-like cell death. *A. tumefaciens* (strain GV3101) carrying empty vector, 35S-*OsHsp18.0*, 35S-*Cf-9*, or 35S-*avr9* were diluted to OD_600_=0.2 and infiltrated into the leaves of *N. benthamiana*. This representative leaf was photographed at 4 d post infiltration. Tomato *R* gene *Cf-9* confers resistance to the fungus *Cladosporium fulvum* carrying the *avr9* avirulence gene. It was previously reported that the interaction of *avr9* and *Cf-9* gene products results in widespread hypersensitive response. Co-infiltration of 35S-*Cf-9* and 35S-*avr9* was served as a positive control here. As expected, the first signs of cell death were observed as water soaking in the infiltrated areas at 2 d post infiltration, and the HR cell death became apparent at 3 d post infiltration. No hypersensitive response was observed when the negative control (empty vector) or OsHsp18.0 was transiently overexpressed, indicating that expression of *OsHsp18.0* could not induce cell death.


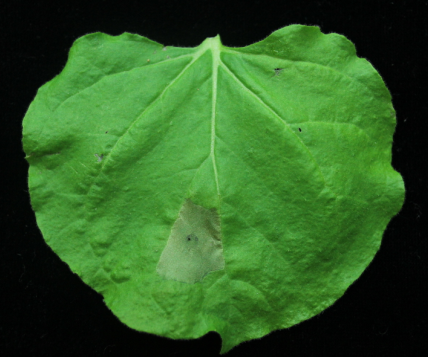


**Empty vector**

**OsHsp18.0**

**Cf-9/Avr9**

**Figure S6.** Comparisons of fresh weight, seedling height, and leaf length between the wild type and *OsHsp18.0* transgenic plants at five-leaf stage. Mean values ± SD from three independent experiments are shown (n = 8). The asterisks indicate that a significant difference in expression was detected between transgenic lines and the wild type.

*
